# A Case of Strangulated Hernia

**Published:** 1894-12

**Authors:** A. L. Fuller

**Affiliations:** Resident Physician Harris County Hospital, Houston


For Texas Medical Journal.

### A CASE OF STRANGULATED HERNIA.

BY DR. A. L. FULLER,
Resident Physician Harris County Hospital, Houston.

THE PATIENT, a colored man, aged about 45, was brought into the St. Joseph's hospital, suffering from severe pain in the neighborhood of the umbilicus. Pain came on suddenly, about two hours previous to admission. He was semi-collapsed, with rapid weak pulse. He gave a history of rupture for over twenty years. Examination revealed a tumor in the right half of the scrotum, which evidently came from the abdomen, and was tense, tympanitic, irreducible and gave no impulse on coughing. On the same side was a suppurating bubo, and on the penis were found two chancroids. Having tried taxis and failed to reduce the hernia, I called in Drs. Hendrick and Morris, and hnving everything ready for herniotomy, we placed the patient fully under an anesthetic and again tried taxis. Failing again to reduce the hernia, we proceeded to the more serious operation.

After thoroughly cleaning the parts, the chancroids and bubo were cauterized with nitric acid. The canal was laid open and the sac being exposed, was incised, revealing about two feet of small intestine and the cæcum with the appendix, which was about six inches in length. The whole was contained in one complete sac. The constriction was relieved by cutting, and the gut pulled down and examined at the seat of the restriction. Being found thoroughly healthy, it was returned into the abdomen. The sac was then ligated and cut off, the stump being returned into the abdomen and fixed to the wall above the opening by the ligatures, and the canal was closed in the usual manner. Antiseptic precautions were observed during the operation and a dressing applied after a drainage tube had been inserted into the scrotum through an opening in its most dependent part.

For the next three days salines were given in such doses as to give free purgation, and, with the exception of slight tympany, the patient progressed favorably, his pulse ranging from 110 at first to 85 on the third day, and his temperature ranging from 101 to normal in the same time. On the third day the dressings were changed, and the wound appeared quite healthy. The bubo was again cauterized, as it did not appear healthy, and the dressings were reapplied. From this on to the 12th day, everything progressed favorably, with the exception of one or two superficial stitch abscesses. On the twelfth day, however, the dressings were changed and the wound found open down to the canal, presenting a surface typical of a chancroid, and the cord was seen exposed in the canal. I at once cauterized the wound and the bubo, making sure that this time all infected surface should be reached, dusted the wound with iodoform and calomel, equal parts, plugged it with gauze, and left it to granulate up. For some days the wound made no progress, until on the eighteenth day, though I could get no history of syphilis, I decided on trying antisyphilitic remedies. Under this treatment the wound soon healed, and on the 25th day the patient was discharged, with an injunction to wear a truss for some months. This he has not done, but at present, three months after the operation, he is well and at work. The site of the canal is occupied by a mass of hard cicatricial tissue, which, I believe, will effectually close the canal, and prevent any re-descent of the hernia.

The chief point of interest in this case is the fact of the infection of the wound by chancroidal virus having prevented union by first intention, and made it necessary to allow the wound to granulate, thus producing a somewhat rough modification of McBurney's operation for a radical cure. It was at the time, an extremely annoying incident, but so satisfied am I with the result, that in future, should I be called on to do any operations for a radical cure, I shall choose McBurney's operation as the one calculated to give the best results as to the *certainty* of success; for while it unquestionably has the disadvantage of being slower than other methods in healing, this is compensated for by a much greater probability of success; this uncertainiy of a permanent cure being the weak point of all such operations.

Another point of interest is the fact of cæcal hernia being enclosed in a complete sac, as most of the authorities mention this as being a rare condition. It seems to me, however, that this condition should be present more often than not with hernia of the cæcum, as the cæcum would hardly be likely to form part of a hernia, except in those cases in which it is completely invested with peritoneum and has a mesentery, unless it were dragged through the canal by a predecending part of small intestine, which would produce the same condition, as, being dragged down in this way, it would necessarily carry a covering of peritoneum with it. Of course, it is quite possible that the cæcum might descend behind the peritoneum, but I cannot help thinking that this is a less common method of descent than the one above mentioned.

